# Hyperglycemia and Angiotensin-Converting Enzyme 2 in Pulmonary Function in the Context of SARS-CoV-2 Infection

**DOI:** 10.3389/fmed.2021.758414

**Published:** 2022-01-13

**Authors:** Jose R. Vargas-Rodriguez, Idalia Garza-Veloz, Virginia Flores-Morales, Jose I. Badillo-Almaraz, Maria R. Rocha-Pizaña, José J. Valdés-Aguayo, Margarita L. Martinez-Fierro

**Affiliations:** ^1^Molecular Medicine Laboratory, Unidad Academica de Medicina Humana y C.S., Campus UAZ Siglo XXI, Universidad Autonoma de Zacatecas, Zacatecas, Mexico; ^2^Laboratorio de Sintesis Asimetrica y Bioenergetica, Ingenieria Quimica, Unidad Academica de Ciencias Quimicas, Campus UAZ Siglo XXI, Universidad Autonoma de Zacatecas, Zacatecas, Mexico; ^3^Escuela de Ingenieria y Ciencias, Tecnologico de Monterrey Campus Puebla, Puebla, Mexico

**Keywords:** ACE2 glycation, non-enzymatic glycation, hyperglycemia, COVID-19, SARS-CoV-2

## Abstract

Since the appearance of the severe acute respiratory syndrome coronavirus (SARS-CoV) in 2003 in China, diabetes mellitus (DM) and hyperglycemia in patients infected with SARS-CoV, represent independent predictors of mortality. Therefore, metabolic control has played a major role in the prognosis of these patients. In the current pandemic of coronavirus disease 19 (COVID-19), multiple studies have shown that DM is one of the main comorbidities associated with COVID-19 and higher risk of complications and death. The incidence and prevalence of COVID-19 complications and death related with hyperglycemia in patients with or without DM are high. There are many hypotheses related with worse prognosis and death related to COVID-19 and/or hyperglycemia. However, the information about the interplay between hyperglycemia and angiotensin-converting enzyme 2 (ACE2), the critical receptor for severe acute respiratory syndrome coronavirus 2 (SARS-CoV-2), in the context of SARS-CoV-2 infection, is almost null, but there is enough information to consider the possible participation of hyperglycemia in the glycation of this protein, unleashing a pool of reactions leading to acute respiratory distress syndrome and death in patients with COVID-19. In this document we investigated the current evidence related with ACE2 as a key element within the pathophysiological mechanism related with hyperglycemia extrapolating it to context of SARS-CoV-2 infection and its relationship with worse prognosis and death for COVID-19.

## Introduction

The current coronavirus disease 19 (COVID-19) pandemic caused by Severe Acute Respiratory Syndrome Coronavirus 2 (SARS-CoV-2) has accumulated, to date (August 2021), more than 205 million confirmed cases and 4,330,703 deaths worldwide (1), representing a lethality of 2.1%. In Mexico there are more than 3,200,000 confirmed cases and 258,856 deaths (2), with a lethality of 8.0%, this being well above the international average. From the total of fatal COVID-19 cases in Mexico, 44.4% of patients had underlying hypertension and 36.8% had diabetes mellitus (DM) (2).

Worldwide, metabolic diseases have become a great challenge and an important public health problem. One of them, and most prevalent, is which represents 9.3% of the world population of adults over 20 years old (3). In Mexico 10.3% of the population over 20 years old has type 2 diabetes (4).

DM is a set of metabolic diseases characterized by hyperglycemia due to defects in the secretion of insulin, its action or both (5). However, there are other causes of hyperglycemia. In hospitalized patients or outpatients, hyperglycemia is a common finding in the emergency services, representing up to 40% of hospital admissions for this cause (6).

Since the appearance of Severe Acute Respiratory Syndrome (SARS) in 2003 in China was observed that diabetes and hyperglycemia in these patients are an independent predictor of morbidity and mortality, and metabolic control plays a major role in the prognosis of these patients (7, 8). In this current pandemic, multiple studies have also shown that DM is one of the main comorbidities associated with COVID-19, and hyperglycemia due to this and other causes has been associated with higher risk of complications and death (8–11).

**Figure 1:**
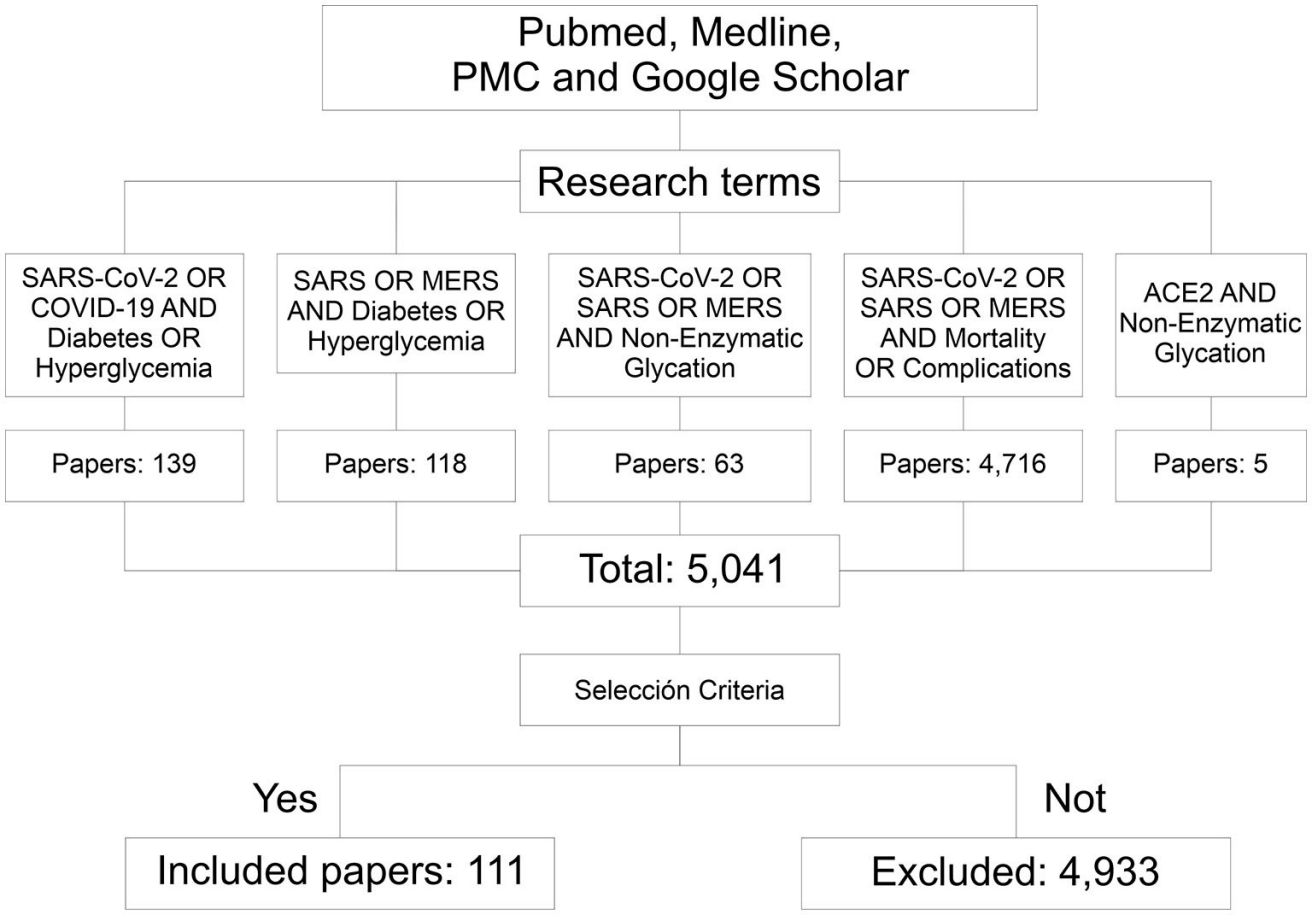
Flowchart of the information searching method. Through the research terms 5,041 papers related to this topic were identified. According to the inclusion criteria, 111 of them were considered for this documentary research. PMC, PubMed central; SARS, severe acute respiratory syndrome; CoV, coronavirus; MERS, meddle east respiratory syndrome.

Some pathophysiological mechanisms have been proposed that could cause this increase in severity and mortality in hyperglycemic patients, with or without DM, in respiratory diseases such as those caused by Severe Acute Respiratory Syndrome Coronavirus (SARS-CoV), Meddle East Respiratory Syndrome Coronavirus (MERS-CoV) and Severe Acute Respiratory Syndrome Coronavirus 2 (SARS-CoV-2). They include, chronic inflammation mechanisms, an abnormal immune response, coagulation alterations and/or damage to pancreatic cells (8, 12). Another possible mechanism is the non-enzymatic glycation of angiotensin-converting enzyme 2 (ACE2) (13), since its expression has been observed to decrease in patients with DM and it could predispose to greater severity and death in respiratory diseases (11) such as COVID-19 (13, 14). There is no evidence demonstrates the role of hyperglycemia on the expression of glycosylated ACE2 and/or on the non-enzymatic glycation of ACE2, which could impact its expression in the cell surface and its binding to SARS-CoV-2 spike protein (15). To describe and discuss these mechanisms and topics, in this document we carried out a search of articles in PubMed, Medline, PMC and Google Scholar databases till October 3, 2020, using the keywords “SARS-CoV-2,” “COVID-19,” “SARS,” “MERS,” “Hyperglycemia,” “Diabetes,” “Mortality,” “Complications,” “Non-enzymatic glycation,” and “ACE2” with interposition of the Boolean operator “AND.” More than 5,000 articles were screened using selection criteria such as being related to SARS-CoV, MERS-CoV or SARS-CoV-2, as well as DM and/or hyperglycemia and its complications, in addition to ACE2 and non-enzymatic glycation. Finally, 111 studies were included in this article (Figure 1).

## Hyperglycemia, Diabetes, and COVID-19

### Epidemiology

The incidence and prevalence of complications and death from COVID-19 related to hyperglycemia in patients with or without DM are still high around the world. From the six main countries with the highest prevalence of DM, four are also among the six countries with the highest mortality from COVID-19: United States, India, Brazil and Mexico (Table 1) (1, 16). Although currently there are many studies related to this topic, especially in recent months, there have been similar reports since the SARS and MERS outbreaks in 2003 and 2012, respectively (7, 12, 17); however, the pathophysiology implicated in worsening evolution and increased risk of death in patients with uncontrolled hyperglycemia is not yet well-known.

**Table 1:** Number of diabetes mellitus and COVID-19 related deaths.

**Country**	**Place**	**Diabetes mellitus-related deaths (2019) (16)**	**Place**	**COVID-19-related deaths (August, 2021) (1)**
China	1	116,400	47	4,848
India	2	77,000	3	429,669
United States	3	31,000	1	618,813
Pakistan	4	19,400	31	24,187
Brazil	5	16,800	2	565,748
México	6	12,800	4	258,856

*Summary of countries with the highest prevalence of DM related deaths and their comparison with the main countries with the highest COVID-19 mortality*.

*DM, diabetes mellitus; COVID-19, coronavirus disease 2019*.

According to the meta-analysis by Bolin Wang et al. during the COVID-19 pandemic, the odds related with the main comorbidities associated with fatal outcomes have been calculated for DM in 2.47 (*P* < 0.001), 2.29 for hypertension (*P* < 0.001), 2.93 for cardiovascular diseases (*P* < 0.001), 3.89 for cerebrovascular disease (*P* < 0.002), and 5.97 for chronic obstructive pulmonary disease (*P* < 0.001) (18), increasing the risk of severity by 5.65 times mainly due to organ failure, and the risk of death 1.1 times compared to the general population (12, 19).

### SARS-CoV-2 and COVID-19

Coronaviruses family belonging into to order of *Nidovirales*, suborder of *Coronavirineae*, and, according to the current nomenclature of the International Committee on Taxonomy of viruses, we can divide them into alpha, beta, gamma and deltacoronaviruses (20). They are viruses wrapped in a single positive sense strand of RNA ~26–32 kb in size, being the largest known genomes for an RNA virus (21) and which are responsible for various respiratory, gastrointestinal and central nervous system diseases in humans and other animals (22). The Human Coronaviruses (HCoV) HCoV-OC43, HCoV-229E, HCoV-NL63, and HCoV-HKU1 generally cause mild respiratory infections, but outbreaks of SARS-CoV, MERS-CoV (22) and, recently, SARS-CoV-2 (23) have shown the ability of these viruses to pass from one species to another and their high pathogenic potential (22). The term coronavirus refers to the appearance of the virions observed under an electron microscope: spine-shaped membrane projections are seen that give it the appearance of a crown (24, 25). All coronaviruses share some similarities in the way they structure and express their genome: they have sixteen non-structural proteins named nsp1–nsp16 that are encoded by the open reading frame (ORF) 1a/b at the 5' end, followed by structural (S), envelope (E), membrane (M) and nucleocapsid (N) proteins that are encoded by another ORF at the 3' end (21).

Entry of the coronavirus into the host cell is a complex process mediated by binding of the viral S glycoprotein binding that contains two domains, S1 and S2, in each monomer. It is a homotrimer with each monomer formed by 1,281 amino acids (26). This homotrimer plays a critical role in infection (22), and together with its interaction with ACE2, promotes fusion of the viral and cellular membranes allowing internalization of the virus for its intracellular replication (20, 27) and causing cell viral infection mainly in the lung, where ACE2 is mainly expressed in type 2 pneumocytes (28) and causing a decrease in the number of ACE2 receptor molecules on the cell surface (15).

COVID-19 had its beginning in the city of Wuhan at the end of 2019, initially appearing as a form of atypical pneumonia caused by a new type of coronavirus, classified initially as 2019-nCoV, and quickly spreading through different provinces of China and other cities of the world in <30 days, putting all health systems on alert (27). This new coronavirus mainly invades the respiratory tract and lungs; severe cases rapidly progress to ARDS, septic shock and multiple organ dysfunction (29), especially in patients with chronic comorbidities such as hypertension, cancer, DM, cardiovascular diseases and acute renal failure, increasing the risk of mortality (30).

### Diabetes and Stress-Induced Hyperglycemia

Diabetes is defined by the American Diabetes Association (ADA) as a group of metabolic diseases characterized by hyperglycemia due to defects in the secretion of insulin, its action, or both (5). In México, 10.3% of the population over 20 years of age suffers from it and it has maintained a constant increase in recent decades (4).

Types 1 and 2 diabetes mellitus are the most frequent of this heterogeneous group of diseases and their symptoms and prognosis can vary considerably. The main difference between these two conditions is that in type 1 diabetes there is destruction of β-cells of the pancreas, generating an absolute deficiency of insulin. In type 2 diabetes, there is a progressive decrease in insulin secretion, with resistance to this hormone (31, 32). These main differences are important to define the most appropriate treatment plan, although sometimes in some individuals it is difficult to define the type of diabetes (32, 33).

Stress-induced hyperglycemia is defined as any elevation of serum glucose at the time of hospital admission in patients with or without previously diagnosed DM (34). This phenomenon has been studied since the 1800s, observed in studies on hypoxia and hyperglycemia and their effects on the body in animal models (35). Stress-induced hyperglycemia is a common problem in Intensive Care Units (ICUs) (36–38), although it is mostly observed in patients with catabolic diseases such as head trauma, burns, sepsis and myocardial infarction (39, 40). It can also occur in patients without a record of metabolic diseases or with previous normal glucose levels (37, 38). This is due to increased hepatic gluconeogenesis and peripheral insulin resistance due to the inhibition of insulin-dependent glucose transporters (GLUT4) by the release of counter-regulatory hormones such as glucagon, cortisol, growth hormones and catecholamine, tumor necrosis factor alpha (TNF-α), and interleukins 1 and 6 (IL-1 and IL-6) (37). The evidence indicates that hyperglycemia >135 mg/dl in patients admitted to ICUs is an independent factor of mortality (41, 42), and that the duration and degree of hyperglycemia is related to an increase in complications, days of hospital stay and death (40, 43). On the other hand, glycemic control within normal ranges has been related to a decrease in morbidity and mortality in ICU patients, although there is still controversy regarding the appropriate levels, since strict glucose control can lead to potentially dangerous hypoglycemia (42). For these reasons, it is important to know and analyze the lung function in patients with hyperglycemia and/or DM and the changes that occur because of these alterations.

### The Lung Function in an Environment of Hyperglycemia

#### Lung Function in Diabetes Mellitus

The implications of DM and hyperglycemia on lung function are poorly understood (44). The lung is generally ignored in the list of target organs in patients with diabetes, but there is increasing evidence of pulmonary vascular damage (44). On the other hand, thickening of the alveolar epithelium and basal lamina of the pulmonary capillaries (44, 45) is a common finding in histopathological analyses of patients with DM, in addition to various observations of alterations in static volumes and in alveolar-capillary diffusion both in type 1 and type 2 diabetes (45).

Changes in spirometry tests have been observed in patients with type 1 and type 2 diabetes, such as in total lung capacity (TLC), vital capacity (VC) and forced expiratory volume in 1 second (FEV1), as well as reduced diffusion capacity for carbon monoxide (DLCO); the proposed mechanisms include increased distance of gas diffusion due to thickening of the basal layer of the pulmonary capillaries and/or the alveolar-capillary matrix (45). Another aspect related to lung capacity is a decrease in the capacity of inspiratory muscles, either due to a restriction of the inspiratory muscles and diaphragm force or to a decrease in muscular resistance when strength is preserved (45). Hyperglycemia and elevated glycosylated hemoglobin (HbA1c) levels in patients with DM are related to these functional alterations in the lung (45), but hyperglycemia has been associated in patients without diabetes as an important predictor of pulmonary dysfunction (45, 46). This fact is generally not related to the biochemical markers of glycemic control, but there is a positive relationship between the decrease in lung function and the duration of hyperglycemia (45).

#### Diabetes Mellitus and Hyperglycemia: Over-Added Lung Infectious

In recent decades, immunological alterations in patients with DM and the risk of suffering from severe infections have been widely studied. The greater susceptibility to infections of patients with chronic hyperglycemia may be due to various abnormalities in the adaptive and innate immunity response and alterations in vascularity that facilitate the colonization of various organisms (47). Several studies have confirmed the increased risk of severity and mortality in those patients with community-acquired pneumonia in addition to the risk of severe bacteremia due to alterations in the response of neutrophils influenced by hyperglycemia (48) due to alteration of opsonization by binding of glucose to the biochemically active site of the third component of complement C3m inhibiting its binding to the microbial surface (49, 50). Hyperglycemia-induced polymorphonuclear (PMN) dysfunction has been related (in patients with and without DM) to a significantly reduced chemotaxis and with increased complications in these patients due to increased secretion of IL-1, IL-6 and TNF-α (51). It has been suggested that HbA1c >8% may alter the correct performance of CD4 lymphocytes (52) and also chronic hyperglycemia promotes monocytes activation and the synthesis of proinflammatory cytokines, aggravating inflammation and increasing lung damage worsening the clinical condition (20) in addition to increasing in oxidative stress (OS) and the reduced availability of nitric oxide (NO) for reactive oxygen species (ROS) that activates transcription of proinflammatory factors and increasing expression of TNF-α and IL-6 (20, 53).

These mechanisms are assumed by previous studies related to other viral infections in patients with DM. The decrease in immune response in DM is reflected in the proliferative response of lymphocytes, particularly in those patients without adequate glycemic control, leading to leukopenia that has been associated as a predictor of severity in COVID-19 patients (20, 54).

Respiratory diseases in patients with DM are not among the top five main causes of death related to this cause, but there is a direct relationship between these pathologies and serious complications and increased mortality in those patients (48, 52); it is also one of the main infections related to DM (49).

Currently, SARS-CoV-2 infection is a major public health problem and one in which higher mortality has recently been observed in hyperglycemic patients with and without a history of DM, so analysis of the pathophysiological processes involved in this disease is vital to understand its course, prevent its complications and reduce mortality from this cause.

Uncontrolled diabetes with serum glucose >180 mg/dl with HbA1c >9% has been associated with a 60% increase of risk of in-hospital over-aggregated pneumonia (12, 17, 55). Some studies even refer to a high risk from an HbA1c >8% of a higher prevalence of leukocytosis, neutrophilia, elevated C-reactive protein, procalcitonin, aspartate transaminase (AST) and D-dimer in COVID-19 patients (17).

In viral infections, susceptibility may be due to alterations in innate, humoral and cell-mediated immunity (20). DM is very common among hospitalized patients with COVID-19 (13, 15) and has emerged as the major risk factor significantly related to increased risk of developing severe forms of COVID-19 (56–58) has a significant impact on the treatment (13) and negatively influences clinical outcomes (15, 59). Although the role of DM in the worsening of COVID-19 is not yet clear, in infections by other viruses such as in the SARS-CoV outbreak in 2003, Influenza A H1N1 in 2009 (7) and MERS-CoV in 2012, it was observed that patients with DM had a RR of 7.2–15.7 for severe complications, greater risk of hospitalization and admission to the ICU and 35% higher mortality than the general population (12).

As already mentioned, during the COVID-19 pandemic, two of the main comorbidities associated with fatal outcomes have been DM and chronic obstructive pulmonary disease as is reported in Bolin Wang et al. meta-analysis (18), with an increase in risk of severity 5.65 times mainly due to organ failure, and the risk of death 1.1 times compared to the general population (12, 19). In patients with DM and chronic obstructive pulmonary disease, hyperglycemia has been related to greater complications due to an increase in proinflammatory cytokines and fibrosis of the extracellular matrix of the lung parenchyma (60). Lung fibrosis was observed in the SARS-CoV outbreak in 2002. In the acute phase of SARS-CoV infection, lung damage and subsequent edema, alveolar shedding of epithelial cells and the deposition of hyaline material in the alveolar membranes reduce the efficiency for gas exchange. During the next phase of infection (weeks 2–5), the lungs show signs of fibrosis due to the deposition of fibrin and infiltration of inflammatory cells and fibroblasts close to the epithelial cells in the alveolar spaces. In the final stage (weeks 6–8), the lung tissue becomes fibrotic with collagen deposits, and epithelial cell proliferation in observed in alveoli and interstitial spaces (61).

The collision between two major current public health problems, the high worldwide prevalence of type 2 diabetes and the COVID-19 pandemic, leads us to a difficult outlook given the high rate of complications and risk of death in those patients (62). This association between diabetes and a worse prognosis in this viral infection is related to hyperglycemia, which is decisive and should always be taken into account for adequate control of viremia and inflammation, thus preventing and increase in morbidity and mortality (63), but keeping in mind also hat too strict glycemic control can lead to dangerous hypoglycemia that also increases the risk of mortality (62, 63).

### The Role of Angiotensin-Converting Enzyme 2

#### ACE2 in Respiratory Diseases

ACE2 is 40 kb in size; its encoding is located in chromosome Xp22 and it contains 18 exons (64–68), many of which resemble those of ACE, and like the latter, it also belongs to the M2 family of metalloproteases (69). Human ACE2 comprises 805 amino acids, of which 40% are identical to the sequence of ACE (66) and it only has one catalytic domain (65). It is an integral type I membrane glycoprotein with its amino terminus and its catalytic domain toward the extracellular space, where it can metabolize circulating peptides (65).

ACE2 also provides a direct binding site for the S protein of coronaviruses (64, 70–74); to activate a receptor of the cell host, the receptor binding domain (RBD) in S1 undergoes hinge-shaped conformational movements that hide or transiently expose the determinants of receptor binding (75, 76).

Acute respiratory distress syndrome (ARDS) is the most severe form of respiratory diseases involving acute lung injury, characterized by pulmonary edema, severe hypoxia and accumulation of inflammatory cells (77); this can be caused by multiple diseases such as sepsis, aspiration in ICU patients, pancreatitis and respiratory diseases such as influenza, SARS-CoV (67, 68, 78), bacterial pneumonia (68) and, currently, SARS-CoV-2 (23).

Since ACE, Angiotensin II (Ang II) and AT1R receptors seem to promote acute lung injury, ACE2 and AT2R receptors seems to have a protective role in pathogen-induced acute lung injury due to the negative regulation of Ang II (78). Since the SARS-CoV outbreak, the pivotal role of ACE2 as a protective factor has been described in COVID-19 (68); however, in spite of ACE2 being the receptor for these viruses, there is a decrease in the abundance of these proteins on the surface, their expression and their enzymatic action by the binding of coronavirus protein S. This is due in part to their detachment and internalization (68), with the consequent increase of Ang II, leading to more severe pathology of ARDS and greater acute lung injury (68, 78).

In ARDS, the pulmonary circulation and Renin-Angiotensin System (RAS) play a very important role; this is where ACE2 becomes notorious since low expression leads to an increase in capillary permeability that triggers pulmonary edema, possibly mediated by an increase in Ang II and its stimulation on AT1R receptors, where other mediators such as eicosanoids, prostaglandins E2 and I2 that regulate changes in capillary permeability also participate (78).

**Figure 2:**
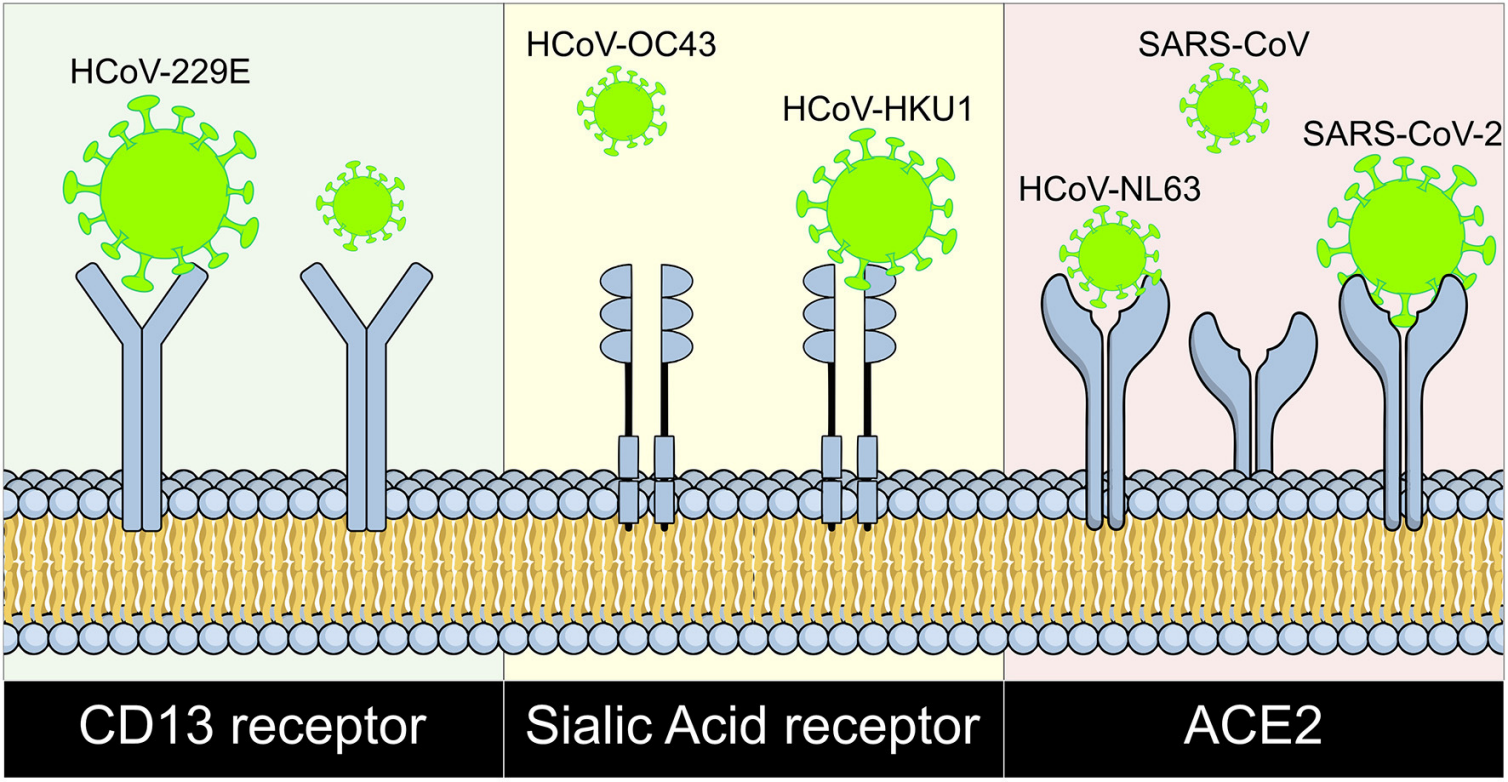
Coronaviruses and their cell surface receptors. The cell receptor for each human coronavirus known is shown. CD13 serves as receptor for HCoV-229E, HCoV-OC43, and HKU1 bind to sialic acid receptors; and ACE2 works as receptor for HCoV-NL63, SARS-CoV, and SARS-CoV-2. HCoV, human coronavirus; SARS, Severe Acute Respiratory Syndrome; CoV, Coronavirus; ACE2, Angiotensin Converting Enzyme 2; CD13, Cluster of differentiation 13.

It is well-known that the respiratory tract is the main site of coronavirus infection (79, 80) causing some respiratory diseases like bronchiolitis and pneumonia infecting the respiratory epithelium, especially in SARS-CoV infection where a viral susceptibility of the epithelium has been observed (68, 79), locating viral RNA both in the air ducts and the alveoli. The different coronaviruses that participate in human infections have different receptors, so HCoV-229E uses CD13 as receptor (80), HCoV-OC43 and HKU1 use Sialic Acid receptors (81, 82), and NL63, SARS-CoV, and SARS-CoV-2 use ACE2 as their receptor (Figure 2). ACE2 interacts with the glycoprotein S of viruses through its extracellular portion that includes an α-helix, lysine-353 and proximal residues of the amino terminal of β-sheet 5 that has high binding affinity with protein S (80); it is mainly well-differentiated apical epithelial cells that are affected (68), which supports viral entry and replication (80).

In the lung, ACE2 is located together with cholesterol and sphingolipids in microdomains in the membrane and its expression level is passively correlated with the differentiation status of airway epithelial cells (68).

The influence of inflammatory cells and their related proinflammatory cytokines in the acute inflammatory phases of the lung has been proposed as one of the initial steps for the development of fibrosis leading to a chronic inflammatory phase (77). ACE2 modulates neutrophil infiltration by inhibiting Ang II/AT1R axis. TGF-β1 is the main and most powerful pro-fibrotic cytokine that acts under regulation of Ang II (68, 77). TGF-β1participates in the conversion of fibroblasts to myofibroblasts and the accumulation of collagen (77), so that the regulation of synthesis contributes to the anti-fibrogenic effect of the ACE2/Ang 1-7/Mas axis, which has been shown to block the pro-fibrotic signaling of Ang II, endothelin-1 and other fibrogenic molecules (68).

The generation of ROS also plays an important role in pulmonary fibrosis, and nicotinamide adenine dinucleotide phosphate (NADPH) oxidase has been proposed as the main source of ROS in pulmonary fibrosis. ROS can also activate small GTPases and RhoA that regulate the migration of type I collagen fibers in the extracellular matrix (68).

Evidence indicates that Ang II by binding to its AT1R receptor induces NADPH and initiates deposits in the extracellular matrix, thus Ang II is a profibrotic mediator that normally induces the migration of pulmonary fibroblasts and the synthesis of collagen (68), but also induces the production of ROS including hydrogen peroxide (H_2_O_2_), hydroxyl radicals (^*^OH) and superoxide anions (${\text{O}}_{2}^{-}$) (60). On the other hand, the conversion of Ang II into Ang 1-7 by ACE2 counteracts the action of Ang II in pulmonary fibrosis, so that a depletion of ACE increases collagen deposits in the extracellular matrix, resulting in an imbalance between ACE/ACE2 that has been related to the severe progression of respiratory diseases (68), as seen in patients with chronic obstructive pulmonary disease, where there is greater ACE activity (60). The activation of the AT1R receptor by Ang II plays a critical role in bronchoconstriction, probably also due to the potentiation of endothelin-1 that induces the contraction of bronchial smooth muscle (68). Ang II can also upregulate the expression of endothelial adhesion molecules such as ICAM-1 and VCAM-1, P and L-selectins and their respective ligands on the surface of inflammatory cells, facilitating the retention of leukocytes within the lung (60).

#### ACE2 in COVID-19

**Figure 3:**
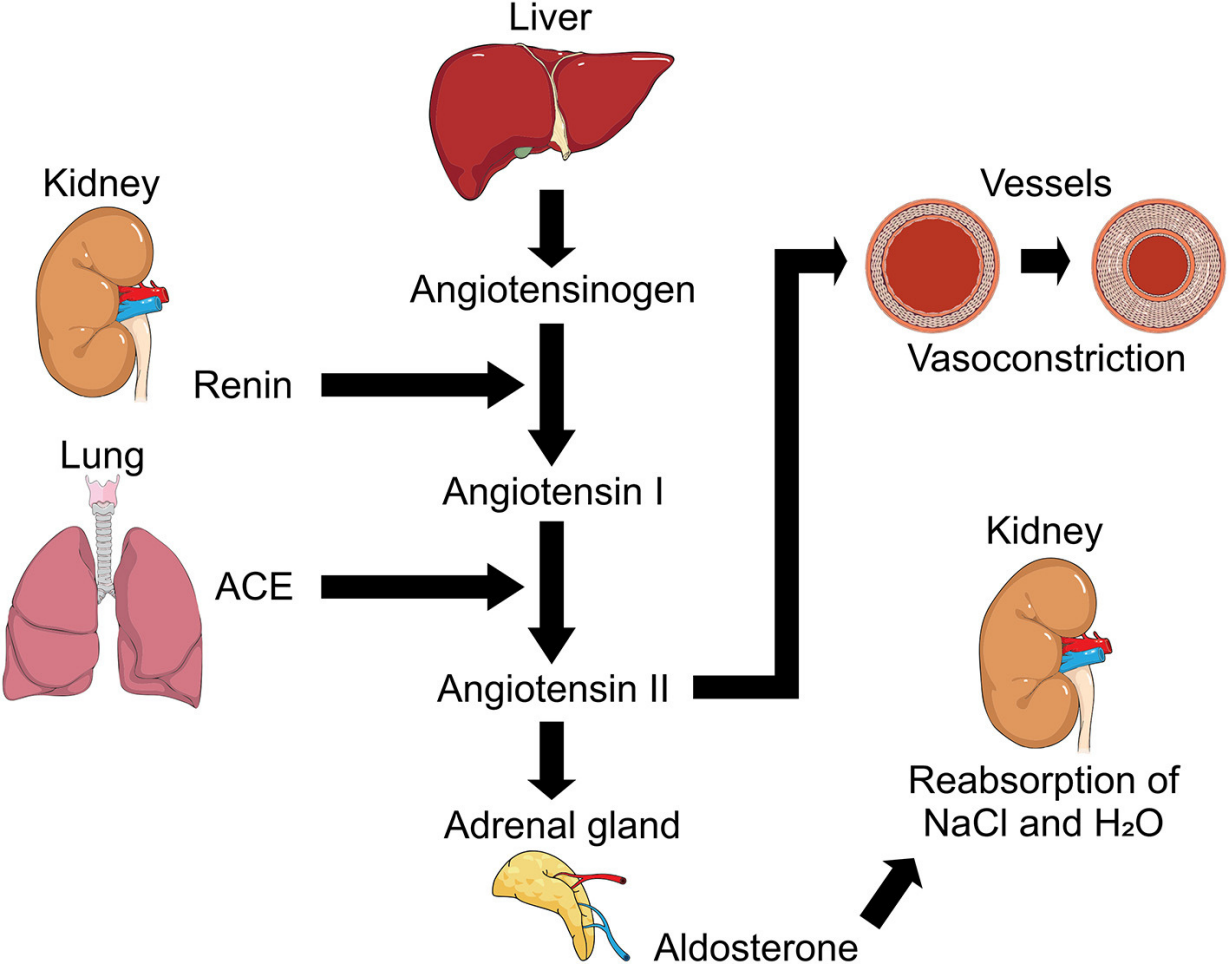
Representation of Renin-Angiotensin-Aldosterone System (RAAS). Regulation mechanism of RAAS and its participation in processes such as the regulation of arterial tension due to its action on vessels causing vasoconstriction with angiotensin II participation, and hydroelectrolytic regulation with the intervention of aldosterone and its action in kidney. ACE, angiotensin converting enzyme; NaCl, sodium chloride (Salt); H_2_O, water.

The renin-angiotensin-aldosterone system (RAAS) is a cardinal system in renal, cardiovascular and respiratory (Figure 3) physiology and pathophysiology (65, 68, 73), maintaining homeostasis in blood pressure and hydroelectric balance (83). Within this complex system, Ang I is converted by the ACE into Ang II which is the main effector substance of RAAS with powerful vasoconstrictor (78), proinflammatory and profibrotic effects (65) mediated angiotensin II receptor type 1 (AT1R) and type 2 (AT2R) (68, 78, 83), but ACE also degrades other substrates such as bradykinin and apelin (78).

Another particular participant is Ang 1-7 which participates in vasodilation, antiproliferation and apoptosis, thus antagonizing the action of Ang II and introducing greater complexity to the RAAS with a homolog of ACE, ACE2. Described in the 2000s (65, 67, 73, 83), this enzyme is capable of cleaving Ang I into Angiotensin 1-9 (Ang 1-9). In addition, ACE2 degrades Ang II to Ang 1-7, which is why it has been suggested that ACE2 acts as a counter-regulator to ACE in the balance between Ang II and Ang 1-7 and participates in the functional clearance of Ang II (65, 74).

Initially, ACE2 expression was reported in the testicles, kidneys and heart, but later studies also demonstrated its expression in other tissues such as the lungs, liver, small intestine and brain (65). Currently, it is known that ACE2 is expressed mainly in the heart, kidneys and small intestine (65, 66, 70, 84) and its abnormal expression has been strongly associated with cardiovascular disease (70). In the respiratory tract, ACE2 is expressed by epithelial cells of the trachea, bronchi, alveoli type I and type II pneumocytes (76), and in vascular endothelial cells, smooth muscle, the brush border of enterocytes, and epithelial cells of renal tubules, among others (64, 84). Since its identification, there are many documented investigation of ACE2 structure and function, its role in various renal and cardiovascular diseases, DM and infectious diseases such as SARS-CoV, and its participation in lung injury (65, 73, 74, 76). Concerning this last point, these viruses cause diffuse alveolar damage with important histopathological changes such as macrophage infiltration, desquamation of epithelial cells and significant apoptosis of pneumocytes (76).

It has been suggested that the S protein of SARS-CoV-2 probably has an affinity for human ACE2 receptors 10–20 times greater than that of SARS-CoV (85), which increases the ability of SARS-CoV-2 to transmit from person to person (86). Structural evidence of ACE2 complex B0AT1, which is a sodium-dependent amino acid transporter present in the intestine (66, 67), suggests that it can bind two S proteins simultaneously (70). There are many similarities between SARS-CoV and SARS-CoV-2; studies have shown that the S proteins of the two viruses are similar, sharing 76.5% of their amino acid sequence (87), while their genome is 79.6% identical (64, 72), with affinity and strong binding to the ACE receptor by RBD (86–88). ACE2 is not only the entry receptor for the virus, but also protects the lung parenchyma (68) and other tissues such as the kidneys and intestine from potential lesions by pathogens, inflammation and fibrosis (64, 66, 72, 78, 89). Greater expression of ACE2 significantly reduces Ang II levels that mediates Ang II-induced acute lung injury (64, 78). ACE2 inhibits apoptosis of lung cells induced by acute lung injury through the up-regulation of Bcl-2 protein, but not of Bcl-2 mRNA (90). However, infections by coronavirus (89, 91, 92) and other viruses such as influenza (74) generate deregulation in the lung protection pathway, which makes them highly lethal (89, 91, 92) since the binding of protein S to ACE2 decreases its expression, in addition to the probable decrease in expression due to glycosylation in hyperglycemic patients (11, 93, 94). The loss of ACE2 expression results in a severe acute respiratory disease due to the loss of its protective factor increasing damage to the parenchyma and pulmonary edema (73, 74, 78, 89, 92), worsening oxygenation and increasing the formation of hyaline membrane and infiltration of inflammatory cells due to deregulation of the pulmonary renin-angiotensin system (RAS) where Ang II is upregulated after the downregulation of ACE2 (64, 74); this apparently occurs without alterations in cardiac contractility or in the tone of the pulmonary vasculature (78). In addition, it has been shown that the SARS-CoV-2 protein S does not use other coronavirus receptors such as aminopeptidases *N*- and dipeptidyl peptidase 4 it binds directly onto the host cell surface to the ACE2 receptors, thus facilitating its entry and replication (64, 91, 95).

The lung appears to be the most vulnerable organ in COVID-19, which may be due to the fact that it has a large surface area that makes it more susceptible to the virus, although various biological factors also participate (91, 95). It has been demonstrated that up to 83% of the pulmonary expression of ACE2 occurs in type 2 pneumocytes (64), which suggests that these cells are the main reservoir for SARS-CoV-2 infection in lung tissue. In addition type 2 pneumocytes that express ACE2 have higher levels of multiple genes related to viral processes, such as those for the viral life cycle, viral assembly and replication of the viral genome, suggesting that type 2 pneumocytes facilitate viral replication in the lung (64, 95).

ACE2 expression is also found in extra-pulmonary tissues, one of greater importance being the small intestine surface where there is also high expression of this enzyme that helps as a co-receptor for nutrients, particularly amino acids (64), so one of the first hypotheses about the onset of the COVID-19 pandemic is intake of food from the Wuhan market with entry of the virus through the enteral epithelium with possible initial fecal-oral transmission. Furthermore, the distribution of ACE2 in various organs may explain the multiorgan failure present in these patients (29, 30, 91). The distribution and degree of expression of ACE2 in some tissues play a decisive role in tropism. In adipose tissue, there is high expression of ACE2, especially in adipocytes, although there is no difference in expression between patients with and without obesity, but a higher number of adipocytes means greater expression of ACE2. Some tumors also express ACE2, such as cervical and pancreatic tumors, both of which express a large number of ACE2 molecules on their cell surface, increasing the risk of infection and complications from SARS-CoV-2 (64).

The role of ACE2 in COVID-19 patients is currently better established and has generated great interest in how ACE2 participates in the pathophysiology of this disease. Lower expression of ACE2 has been observed in elderly men compared to women of the same age (64), which reinforces the observation that elderly men are more susceptible to SARS-CoV-2 infection (66) and develop more severe symptoms and complications of COVID-19 (64, 66), even more so those with associated comorbidities, although other factors such as the immune response of individuals and viral load also participate (64).

### Protein Glycation and Chronic Diseases

#### Chronic Hyperglycemia and Protein Glycation

Protein glycation is a spontaneous (96), non-enzymatic and complex reaction (97) of union between the free amino group of a protein, typically the ε group, and the carbonyl group of a reducing sugar (glucose, galactose, fructose, mannose or ribose) generating a Schiff base (97, 98) after the release of a water molecule; it is easily reversible and all its intermediate metabolites are highly reactive (96). In a process that takes hours, the Schiff base is modified into more stable compounds such as Amadori products formed from aldoses and Heyns-Carson products resulting from ketoses; in a period of days, this modification becomes practically irreversible (96, 99). Later, through a process called a retro-aldol reaction, a series of aldehydes such as glyceraldehyde, formaldehyde and glyoxal are created (96). This process is separate from glycosylation, which is a controlled enzymatic reaction during the synthesis of glycoproteins. It has been observed to occur to varying degrees in young and elderly people but is more associated with sustained hyperglycemia in DM, leading to toxic processes which have a greater impact on health (96).

A main complication of hyperglycemia in patients with and without DM, and its impact on the pathophysiology of various diseases, is the excessive chemical interaction of glucose (100) and other monosaccharaides (101) with the proteins affecting them (99, 102). To a lesser degree, glucose also interacts with lipids and DNA where a union is generated without the need for enzymatic intervention, called non-enzymatic glycosylation or glycation (103). This process depends on the serum glucose concentration and the contact time with proteins, which will be determined by their half-life (99–101), so the degree of glycation will be related to serum glucose levels (99) and the percentage of HbA1c, being greatly increased in patients with DM (104). This reaction was first described in 1912 by the French chemist Louis-Camille Maillard (96, 98, 103, 105) who studied the loss of lysine in preserved foods rich in proteins and glucose (103). Later, others authors studied this reaction, such as Amadori in the 1920s and Hodge in the 1950s; glycosylated hemoglobin was the first molecule related to this reaction discovered in 1955 (96).

Endogenous glycation involves glucose, the most abundant reducing sugar in the body, and the free amino groups of proteins, especially those of amino acids such as lysine and arginine that participates in multiple physiological and pathophysiological processes in patients with and without diabetes. However, patients with diabetes are the most affected because reactions generated by sustained hyperglycemia (that increases and accelerates complications) also generate an excess of reactive carbonyls, increasing carbonyl stress that aggravates inflammation and OS (96). There are several factors that facilitate the endogenous glycation of proteins, such as an increase of body temperature since this reaction cannot occur at 37°C, an alkaline pH which increases the reactivity of free amino groups of proteins, dehydration and the presence of metals in proteins (96). Also, some external factors, such as smoking and exogenous advanced glycation end products (AGEs) from food, induce cell dysfunction and death (106). Dozens of AGEs have been described; the most studied are pentosidine, which has been related to skin aging and some lung diseases, and *N*-ε-Carboxymethyl-lysine (CML), high concentrations of which have been associated with the cellular response to OS (106).

Glycation has a great relevance, both physiological and pathophysiological. In pathological processes, its reaction is faster and more intense, especially in situations with sustained hyperglycemia, affecting proteins by modifications to their structure and function. As an example, has been suggested that DM might promote the ACE2 modifications (glycation), favoring SARS-COV-2 entry (as was seen in cardiomyocytes) (15). However, the greatest biological effects due to excess glycation include the inhibition of regulatory molecules, protein crosslinking and trapping of soluble proteins in the extracellular matrix (104) (generating aberrant protein crosslinks that decrease elasticity) (98). It also promotes increased fibrosis due to glycated collagen, in addition to decreasing susceptibility to proteolysis (96), enzymes inactivation, abnormalities in nucleic acid function and increased immunogenicity in relation to the formation of immune complexes (104). Conventionally, this process has been divided into two stages, one initial or early and the other late or advanced; the fundamental difference between these lies in the reversibility of the initial one, while in the advanced stage it is no longer possible to reverse the process due to the various cascade reactions that are involved which permanently affect the molecules involved (100). In the initial phase of the glycation process, covalent bonds are formed between the aldehyde group of glucose and the free amino groups of proteins (101) which are generally located in the lysine side chains and in the NH_2_- terminal residues of amino acids (100). This only occurs when glucose is in an open chain conformation, exposing its reactive carbonyl group (aldehyde group of glucose), and is what gives rise to the Schiff base which is an unstable aldimine and the first product of glycation; at this point, glycation is still easily reversible (100). The Schiff base can also undergo a slow intramolecular rearrangement that turns it into a more stable product called an Amadori compound, also known as fructosamine (1-amino-1-deoxyketose). An Amadori compound is a metabolite that arises when the glucosyl residue of a Schiff base is transformed into fructosyl by rearrangement of the carbonyl group from carbon 1 to 2 (100, 101). The Amadori compound can be damaged by oxidation to form highly reactive dicarbonyl intermediates called glycotoxins, such as 3-deoxyglucose and CML, which alone can modify proteins. fructosamine levels have been directly correlated with HbA1c levels (102) and it has been observed that alterations attributed to fructosamine predominate in short half-life proteins (100). In late glycation, a series of irreversible reactions takes place, which are collectively called Maillard reactions (101), where the fructosamine is converted into AGEs (103); it occurs in structural proteins, mainly those with a long half-life, although it also affects short half-life proteins, especially when they are retained for a long time (100, 101, 103). These prolonged interactions also affect lipoproteins, in which oxidation reactions are more complex due to the presence of polyunsaturated fatty acids (PUFAs) that are easily oxidizable and in which glycation reactions provide a probable explanation for the increase in atherosclerosis (101).

The formation of AGEs always requires as a prerequisite the formation of fructosamine and the series of reactions that lead to its dissociation products. These reactions lead to the formation of a large number of AGEs, and although oxidation by oxygen free radicals is involved in all these reactions, they can also be derived from the autoxidation of glucose itself, for which CML can be formed by the breakdown of fructosamine or by protein interactions with glyoxal, which contains a dicarbonyl group and can be a product of lipid peroxidation (100). AGEs encompass many reactive species that can be colored yellowish-brown, are fluorescent, and are also cross-linked between different proteins or between different areas of proteins. Some which have been identified are CML, *N*-ε-carboxymethyl-hydroxylysine, 3-(*N*-ε-lysine)-lactic acid, pentosidine, pyrroline and some cross species of the latest (100). Other alternative advanced glycation pathways have been described involving methylglyoxal, another dicarbonyl compound derived from triose phosphates and which is generated inside cells from the metabolism of glyceraldehyde-3-phosphate and the oxidation of ketones; these compounds are highly toxic and are increased in DM, being capable of rapidly modifying proteins and generating AGEs. The metabolism of methylglyoxal is dependent on reduced glutathione, which is reduced when OS increases, conditioning and increasing intracellular toxins and their greater permanence, prolonging their deleterious effects on cells (100).

Triose phosphates such as fructose-1-6-bisphosphate, glyceraldehyde-3-phosphate and dihydroxyacetone phosphate that are the result of the first phase of glycolysis are increased in patients with DM by inhibition of glyceraldehyde-3-phosphate dehydrogenase; these trioses are 100 times more autoxidable and 200 times more glycosylating than glucose, so those are the origin of the enzymatic synthesis of methylglyoxal. In addition, glyceraldehyde-3-phosphate is always in an open chain formation, so it can quickly form AGEs (100).

#### Advanced Glycation end Products and Their Pathogenic Role

AGEs are a heterogeneous group of irreversible products formed by glycation (99, 105) and glycol-oxidation of proteins, nucleic acids and lipids with reduction of sugars. Since the reaction to produce AGEs requires time, it is believed that more of them are accumulated by long half-life proteins (99). Some AGEs can be exogenous, obtained through diet and smoking (99). Receptors for advanced glycation end products (RAGE) are multiligand receptors that are members of the cell surface immunoglobulin superfamily and act as receptors not only for AGEs but also for S100/calgrulins and β-amyloid peptide (98). They are found in smooth muscle cells, macrophages, endothelium and astrocytes (99). They have three receptors (full-length RAGE, *N*-incomplete RAGE and *C*-terminal RAGE), with two isoforms, cleaved RAGE (cRAGE) and endogenous secretory RAGE (esRAGE). the interaction of AGEs and full-length RAGE stimulates the activation of nuclear factor kappa B (NF-κB), increasing the expression of inflammatory cytokines and the production of ROS (98, 99, 107), which increases intracellular OS due to deregulation as a suppression effect or reduction of glutathione and ascorbic acid levels (98).

AGEs can cause tissue damage through two mechanisms, the first one direct, in which phenomena such as decreased resistance of proteins to the proteolytic action of degrading enzymes, crosslinking and trapping of proteins, induction of lipids oxidation occur, and the inactivation of NO happen. The other mechanism is indirect and involves the induction of chemotaxis, stimulating the synthesis of cytokines and the production of extracellular matrix, an increase in plasma procoagulant activity and vascular permeability, and an increase in OS and participation in the DNA mutation index (100).

Formation of AGEs (secondary to hyperglycemia) increases their deposition in the extracellular matrix, altering the functional properties of several important molecules of the endothelial matrix, which leads to cell renewal and growth disorders, with increased of vascular damage. In addition, AGEs induce an increase in TNF-α, IL-1, insulin-like growth factor 1 (IGF-1) and platelet-derived growth factor and suppression of NO synthesis by monocyte-macrophages, and interfere with cell-matrix interactions with modifications of type IV collagen fibers (103), decreasing the adhesion of its binding domains to endothelial cells and reducing the action of thrombomodulin in these cells (100).

The presence of AGEs in the extracellular matrix modifies the functional characteristics of several key molecules. One of the first molecules in which the existence of covalent intermolecular bonds induced by AGEs was discovered is collagen; in type I collagen, molecular aggregation induces the distortion of molecular building of the fibrils (108). The luminal narrowing observed in the blood vessels of patients with DM can be attributed to the subendothelial accumulation of various proteins such as albumin, low density lipoproteins (LDL) and immunoglobulin G (IgG) that are trapped by AGEs in the collagen fibers of the basal membrane by covalent aggregation (103, 108).

AGEs formation in type IV collagen of the basal membrane hinders the lateral association of these molecules in a complex three-dimensional structure (108) and tends to cross-link the fibers in an anarchic way, resulting in increased permeability (103). AGEs have a dose-dependent inactivation effect on NO, altering its vasodilator response and correlating well with the levels of AGEs accumulated in vessels (109, 110).

### Hypothesis Proposal and Discussion

As previously described, there is evidence that shows the multiple complications that occur in DM or as a cause of sustained hyperglycemia in various diseases. The protective role that ACE2 plays in several tissues is well-known, especially in lung parenchyma (78). However, ACE2 is the principal target for SARS-CoV-2 due to the high expression of this enzyme in type 2 pneumocytes and has been determined as the receptor for this virus and other coronaviruses (80, 85).

**Figure 4:**
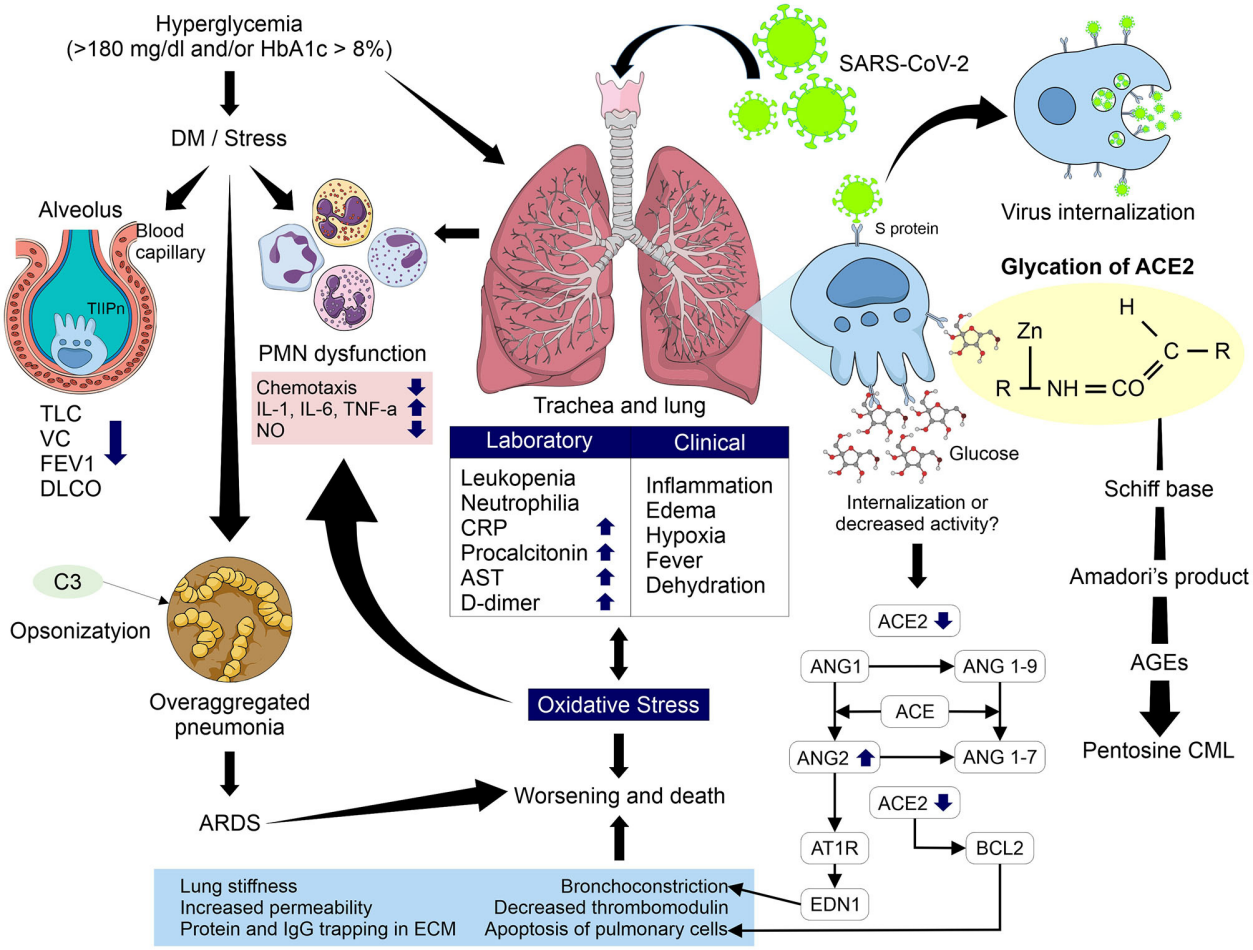
Pathophysiological mechanism involved in glycation of ACE2 in uncontrolled hyperglycemic patients with and without DM and its relationship with complications and mortality due to COVID-19. Glycation may modify the activity of ACE2 causing a several reactions such as increased levels of Ang II, greater interaction with its receptor AT1R, increasing levels of endothelin-1, decreased activity of Bcl-2, and greater interaction of glycation products with type II pneumocytes. These processes increase bronchoconstriction, apoptosis of pulmonary cells, lung stiffness, permeability, and protein and IgG trapping in ECM and decreased thrombomodulin. In addition, an increase in OS, PMN dysfunction and the alterations induced by hyperglycemia and the higher risk of over-aggregated pneumonia in COVID-19 patients could increase the risk of ARDS and therefore of worsening evolution and death. TLC, total lung capacity; VC, vital capacity; FEV1, forced expired volume in 1 second; DLCO, Pulmonary diffusion capacity; PMN, polymorphonuclear; IL, interleukin; TNF-α, tumor necrosis factor alpha; NO, nitric oxide; CRP, C-reactive protein; AST, aspartate aminotransferase; T2Pn, type 2 pneumocytes; ACE, angiotensin converting enzyme; ACE2, angiotensin converting enzyme 2; Ang, angiotensin; AT1R, angiotensin type 1 receptor; AGEs, advanced glycation end products; CML, *N*ε- carboxymethyl-lysine; Bcl-2, B cell lymphoma; ARDS, acute respiratory distress syndrome.

The inherent changes resulting from sustained hyperglycemia, which although it can be a main characteristic of DM but is not exclusive to it, since elevated glucose levels above the normal range but below diagnosis levels can also cause deleterious effects on cells. Some alterations that occur in these cases and that may be related to the increase of complications and death in COVID-19 are alterations in the neutrophil's response, and PMN dysfunction. The consequences include a decrease in chemotaxis and increase in proinflammatory interleukins, mainly IL-1 and IL-6, in addition to TNF-α, which are further increased by OS induced by hyperglycemia, causing a decrease in leukocytes proliferation and an increase in IGF-1 and ROS with decreased production of NO by monocytes/macrophages. In addition, hyperglycemia decrease opsonization by blocking the action of complement C3, increasing the risk and susceptibility to bacterial infections; in COVID-19 patients, an over-aggregated infection such as pneumonia associated with health care can occur. Also, the observation that there is decreased expression of ACE2 in older male adults, and its relationship with more serious complications and death in COVID-19, may play an important role in conjunction with the mechanism studied in this document, that is the glycation of ACE2 and its role as a pivot in the chain of pathophysiological reactions (Figure 4).

Lung fibrosis was observed in the SARS-CoV outbreak in 2002 in the acute phase of infection. Accordingly, is not difficult to infer that similar processes may occur in SARS-CoV-2 infection, due to the great similarities between the two viruses, and that they participate in the worsening of clinical evolution and increased risk of death. A possible explanation could be the severe down regulation or absent expression of ACE2 during the proliferation of active type I pneumocytes during lung fibrosis, that appear to replace the damaged alveolar type II pneumocytes (111), in addition to a decrease in ACE2 representation in cell surface by internalization after its binding to SARS-CoV-2, and/or the possible decrease in enzymatic action by glycation.

Leukopenia, neutrophilia and increased CRP, procalcitonin, AST and D-dimer in SARS-CoV-2 infection (17) will present inflammation by cytokine storm and pulmonary edema that generate hypoxemia and consequent hypoxia, fever and dehydration accompanied by infiltration of macrophages, desquamation of epithelial cells and apoptosis of pneumocytes (76).

As we described in previous sections, ACE2 plays an important role as a lung protector in these types of infection. A decrease in expression of ACE2 because internalization caused by binding of SARS-CoV-2 with the receptors of this enzyme and the probable participation of glycation of ACE2 in uncontrolled hyperglycemic patients could explain the loss of its protective factor due to its internalization and/or inactivation, which are the main effects observed in the proteins affected by glycation. This hypothesis is supported by a recent study carried out by D'Onofrio et al. which demonstrated the enhanced long-term non-enzymatic glycation of ACE2 at the neck domain of dimerization can affect ACE2 oligomerization (15) and it could have relation to the loss of this regulation by ACE2. The consequences may be a possible accumulation of Ang II will increase its vasoconstrictor, proinflammatory and profibrotic effects. Also, a greater interaction with its AT1R receptor will stimulate an increase of endothelin-1, increasing bronchoconstriction, and deregulation of Bcl-2 and lung cell apoptosis, aggravating the clinical evolution and OS entering a vicious circle that is difficult to regulate.

ACE2 is a metalloproteinase with a long half-life, it can be easily glycated in patients with sustained or chronic hyperglycemia, thus favoring the formation of AGEs, mainly pentosine and CML, which have been shown to have a great participation in lung diseases with several effects such as a decrease of thrombomodulin that may be related to the disseminated vascular coagulation proposed as a mechanism involved in COVID-19, and also the elevation of D-dimer.

AGEs can also generate aberrant cross-links due to glycation of type IV collagen fibers, which causes greater vascular permeability, increasing pulmonary edema. In addition, glycation of type I collagen fibers trapps soluble molecules and IgG in the extracellular matrix, decreasing leukocyte proliferation and increasing lung fibrosis with loss of susceptibility to proteolysis reducing the elasticity of the lung parenchyma.

All these pathophysiological alterations together would lead to an ARDS with increasing inflammation due to an uncontrolled increase in cytokines and proinflammatory factors, generating greater pulmonary edema and hypoxia, worsening the clinical evolution of COVID-19 together with the multiple organ failure present in those patients, increasing the risk of death.

Combining the information currently available regarding the pathophysiology of SARS-CoV-2 infection, the negative implications in the body of sustained hyperglycemia related or not to DM, as well as the beneficial effect of glycemic control in COVID-19 patients, indicate the importance of ACE2 in lung protection and its participation in the correct functioning of other tissues, and the deleterious effects of the absence of ACE2. From the glycation mechanism, with the consequent chain of reactions with adverse effects due to the formation of AGEs and the effects of hyperglycemia and glycation *per se*, with this proposal we can make sense of the increase in complications and death risk in COVID-19 and open up a new area of study. Therefore, the glycation of ACE2 may be a possible pathophysiological mechanism of the relationship between diabetes/hyperglycemia and COVID-19, and their relation in worse prognosis and death by this cause.

## Data Availability Statement

The raw data supporting the conclusions of this article will be made available by the authors, without undue reservation.

## Author Contributions

JV-R and MM-F: research, analysis of information, and writing. IG-V: analysis of information and tables and figures construction. VF-M, JB-A, and MR-P: analysis of information. JV-A: analysis of information and writing. MM-F: draft approval. All authors contributed to the article and approved the submitted version.

## Funding

This work was funded in part by the Programa de Apoyos para el Fortalecimiento de Capacidades para el Diagnostico de COVID-19-CONACYT. Grant ID: 314340 (A12 xii. 2020) to MM-F. Edition costs were covered by the Academic Program of Doctorado en Ciencias con Orientación en Medicina Molecular from Academic Unit of Human Medicine and Health Sciences of Universidad Autónoma de Zacatecas.

## Conflict of Interest

The authors declare that the research was conducted in the absence of any commercial or financial relationships that could be construed as a potential conflict of interest.

## Publisher's Note

All claims expressed in this article are solely those of the authors and do not necessarily represent those of their affiliated organizations, or those of the publisher, the editors and the reviewers. Any product that may be evaluated in this article, or claim that may be made by its manufacturer, is not guaranteed or endorsed by the publisher.
